# Supplementary material for: Quantification of purified endogenous miRNAs with high sensitivity and specificity
Source: Nat Commun. 2020 Nov 27;11:6033. doi: 10.1038/s41467-020-19865-9 (PMC7699633; doi:10.1038/s41467-020-19865-9)
Supplement: Supplementary file 1 — Supplementary Information [file 41467_2020_19865_MOESM1_ESM.pdf]

Supplementary Information

Quantification of purified endogenous miRNAs with high sensitivity and specificity

Related to Fig.2d−e

5-UGAGGUAGUAGGUUGUAUAGUU-(U14)-Biotin-3 — let−7a(RNA)
3-TTT**T**TTTTTTTTACTCCATCA-p-5
3-TAT**T**TATTATTACATCATCCA-p-5
3-TTT**T**TATTATCAACATATCAA-p-5

5-UGAGGUAGUAGGUUGUGUGGUU-(U14)-Biotin-3 — let−7b(RNA)
3-TTT**T**TTTTTTTTACTCCATCA-p-5
3-TAT**T**TATTATTACATCATCCA-p-5
3-TAT**T**TATTAGGAACACACCAA-p-5

5-UGAGGUAGUAGGUUGUAUGGUU-(U14)-Biotin-3 — let−7c(RNA)
3-TTT**T**TTTTTTTTACTCCATCA-p-5
3-TAT**T**TATTATTACATCATCCA-p-5
3-TAT**T**TATTTTCAACATACCAA-p-5

5-AGAGGUAGUAGGUUGCAUAGUU-(U14)-Biotin-3 — let−7d(RNA)
3-TTA**T**TATTATTATCTCCATCA-p-5
3-TTT**T**TATTTACTCATCCAACG-p-5
3-TTT**T**TTATTTTCAACGTATCA-p-5

5-UGAGGUAGGAGGUUGUAUAGUU-(U14)-Biotin-3 — let−7e(RNA)
3-TTA**T**TATTATTTACTCCATCC-p-5
3-TTT**T**TTATTATTATCCTCCAA-p-5
3-TTT**T**TATTATCAACATATCAA-p-5

Related to Fig.2f−g

5-UGAGGUAGUAGGUUGUAUAGUU-3 — 22 nt(RNA)
3-TTT**T**TTTTTTTTACTCCATCA-p-5
3-TAA**T**AATAATCATCCAACATA-p-5
3-TAA**T**AATAATCATATCAATTT-p-5

5-UGAGGUAGUAGGUUGUAUAGU-3 — 21 nt(RNA)
3-TTT**T**TTTTTTTTACTCCATCA-p-5
3-TAA**T**AATAATCATCCAACATA-p-5
3-TAA**T**AATAATACATATCATTT-p-5

5-UGAGGUAGUAGGUUGUAUAG-3 — 20 nt(RNA)
3-TTT**T**TTTTTTTTACTCCATCA-p-5
3-TAA**T**AATAATCATCCAACATA-p-5
3-TAA**T**AATAATAACATATCTTTT-p-5

Related to Fig.3

5-UGAGGUAGUAGGUUGUAUAGUU-3 — let−7a(RNA)
3-TTT**T**TTTTTTTTACTCCATCA-p-5
3-TTT**T**TTTTACTCATCCAACAT-p-5
3-TTA**T**TTATTACCAACATATCA-p-5

5-UGAGGUAGUAGGUUGUAUGGUU-3 — let−7c(RNA)
3-TTT**T**TTTTTTTTACTCCATCA-p-5
3-TTT**T**TTTTACTCATCCAACAT-p-5
3-TTA**T**TTATTTTCAACATACCA-p-5

5-Biotin-TTTTTTTTTTTTTTTTTTTTTTTTTTTTTT-3 — Biotin−Poly(T)

Related to Supplementary Fig.4

5-UGGGAUGAGGUAGUAGGUUGUAUAGUUUUA
GGGUCACACCCACCACUGGGAGAUAACUAU
ACAAUCUACUGUCUUUCCUA-3 — Precursor−let−7a−1 (RNA)

Supplementary Figure 1. Oligonucleotides sequences used for the experiments. Sequences of target miRNAs and corresponding DNA probes used in the experiments are presented. Fluorophore labeling positions are indicated in bold. The poly-T DNA used for capturing target miRNAs have 30 Ts.

a TaqMan

Probe \ miRNA	Relative detection (%)				
	let-7a	let-7b	let-7c	let-7d	let-7e
Probe_a	100	0.44	20.89	2.20	3.68
Probe_b	0.19	100	22.48	0.00	0.00
Probe_c	0.09	1.77	100	0.00	0.00
Probe_d	2.59	0.01	1.37	100	0.01
Probe_e	9.88	0.07	7.87	0.09	100

b Quanta

Probe \ miRNA	Relative detection (%)				
	let-7a	let-7b	let-7c	let-7d	let-7e
Probe_a	100	0.27	50.71	2.17	1.58
Probe_b	0.09	100	32.84	0.00	0.00
Probe_c	48.91	27.00	100	0.31	0.56
Probe_d	0.12	0.33	0.07	100	0.00
Probe_e	0.13	0.13	0.13	0.00	100

c miQPCR

Probe \ miRNA	Relative detection (%)				
	let-7a	let-7b	let-7c	let-7d	let-7e
Probe_a	100	12.64	55.52	101.75	122.47
Probe_b	7.78	100	45.46	1.08	0.06
Probe_c	66.40	75.14	100	28.76	1.13
Probe_d	14.84	0.00	0.09	100	0.21
Probe_e	51.07	0.04	20.96	27.57	100

Supplementary Figure 2. Characterization of commercialized qRT-PCR miRNA detection assays. Relative detection (%) of let-7 family miRNAs of (a) TaqMan, (b) Quanta, and (c) miQPCR are presented. The tables are reproduced from Androvic et al. Nucleic acids research 45, e144-e144 (2017). It is clear that false positive rates (off-diagonal elements) of commercial qRT-PCR assays are significantly larger than those of Ago-FISH.

a

	Relative detection (%), threshold: 2% duty cycle				
Probe \ miRNA	let-7a	let-7b	let-7c	let-7d	let-7e
Probe_a	100	6.58	8.20	5.77	11.53
Probe_b	3.28	100	10.30	7.27	8.12
Probe_c	4.87	5.68	100	10.07	4.84
Probe_d	5.22	2.13	14.28	100	2.60
Probe_e	8.31	5.62	5.63	6.87	100

b

	Relative detection (%), threshold: 5% duty cycle				
Probe \ miRNA	let-7a	let-7b	let-7c	let-7d	let-7e
Probe_a	100	0.95	3.09	0.00	2.50
Probe_b	0.00	100	3.40	0.00	0.00
Probe_c	1.27	0.00	100	0.00	0.00
Probe_d	0.00	0.00	5.05	100	0.00
Probe_e	3.26	0.50	0.00	0.00	100

c

	Relative detection (%), threshold: 10% duty cycle				
Probe \ miRNA	let-7a	let-7b	let-7c	let-7d	let-7e
Probe_a	100	0.00	0.00	0.00	0.00
Probe_b	0.00	100	0.00	0.00	0.00
Probe_c	0.00	0.00	100	0.00	0.00
Probe_d	0.00	0.00	0.12	100	0.00
Probe_e	0.00	0.00	0.00	0.00	100

Supplementary Figure 3. Relative detection of Ago-FISH at varying duty cycle thresholds. Positive rates (either true: diagonal, or false: off-diagonal) of let-7 family miRNAs obtained by comparing the average spot numbers. In this approach, the true positive rates (the diagonal elements) are automatically assumed as 100% as in qRT-PCR. The duty cycle thresholds used for the analysis are indicated in the table.

a

Probe \ miRNA	Positive rate (%), threshold: 2% duty cycle				
	let-7a	let-7b	let-7c	let-7d	let-7e
Probe_a	88.83	6.18	7.32	5.21	10.05
Probe_b	2.92	93.88	9.20	6.56	7.08
Probe_c	4.33	5.34	89.24	9.09	4.22
Probe_d	4.64	2.00	12.75	90.19	2.27
Probe_e	7.39	5.28	5.03	6.20	87.09

b

Probe \ miRNA	Positive rate (%), threshold: 10% duty cycle				
	let-7a	let-7b	let-7c	let-7d	let-7e
Probe_a	59.74	0.00	0.00	0.00	0.00
Probe_b	0.00	65.82	0.00	0.00	0.00
Probe_c	0.00	0.00	63.32	0.00	0.00
Probe_d	0.00	0.00	0.08	60.78	0.00
Probe_e	0.00	0.00	0.00	0.00	58.29

Supplementary Figure 4. Estimation of Positive Rates of Ago-FISH at varying duty cycle thresholds. Positive rates (either true: diagonal, or false: off-diagonal) of let-7 family miRNAs were obtained by inspecting duty cycles of DNA probes. We identified a molecule as positive when the duty cycles of all of the three DNA probes are larger than the threshold value. The duty cycle thresholds used for the analysis are indicated in the table.

a

	Relative detection (%), threshold: 2% duty cycle		
miRNA / Probe	22 nt	21 nt	20 nt
Probe _22 nt	100	10.86	4.68
Probe _21 nt	11.02	100	2.98
Probe _20 nt	16.60	12.58	100

	Relative detection (%), threshold: 5% duty cycle		
miRNA / Probe	22 nt	21 nt	20 nt
Probe _22 nt	100	3.76	0.00
Probe _21 nt	4.81	100	1.70
Probe _20 nt	8.78	5.64	100

	Relative detection (%), threshold: 10% duty cycle		
miRNA / Probe	22 nt	21 nt	20 nt
Probe _22 nt	100	0.00	0.00
Probe _21 nt	0.00	100	0.00
Probe _20 nt	0.00	1.00	100

b

	Positive rate (%), threshold: 2% duty cycle		
miRNA / Probe	22 nt	21 nt	20 nt
Probe _22 nt	84.90	9.44	3.87
Probe _21 nt	9.36	86.92	2.47
Probe _20 nt	14.1	10.90	82.69

	Positive rate (%), threshold: 10% duty cycle		
miRNA / Probe	22 nt	21 nt	20 nt
Probe _22 nt	55.40	0.00	0.00
Probe _21 nt	0.00	51.54	0.00
Probe _20 nt	0.00	0.52	52.90

Supplementary Figure 5. Relative detection and positive rates of Ago-FISH for detecting 3'-tailing of miRNAs. (a) Positive rates (either true: diagonal, or false: off-diagonal) of let-7a miRNAs with different 3'-tails obtained by comparing the average spot numbers. In this approach, the true positive rates (the diagonal elements) are automatically assumed as 100% as in qRT-PCR. (b) Positive rates (either true: diagonal, or false: off-diagonal) of let-7a miRNAs with different 3'-tails obtained by inspecting duty cycles of DNA probes. We identified a molecule as positive when the duty cycles of all of the three DNA probes are larger than the threshold value. The duty cycle thresholds used for the analysis are indicated in the table.

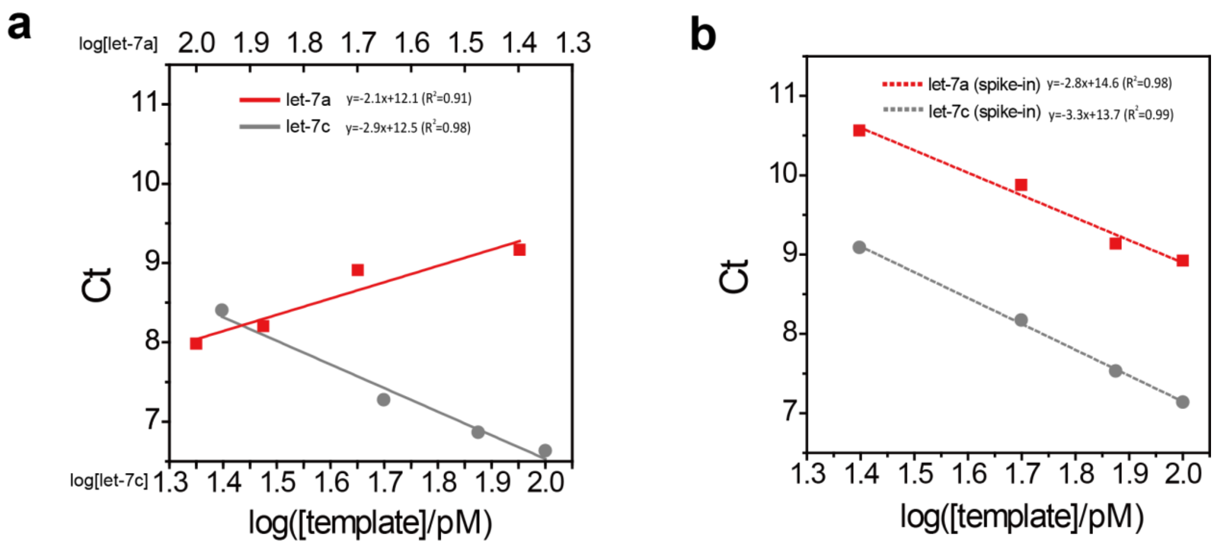

Supplementary Figure 6. Standard curves of qRT-PCR. (a) The Ct values are plotted at varying combination of concentrations of synthetic let-7a and let-7c miRNAs. (b) miRNA spike-in experiments. Varying amount of synthetic let-7a and let-7c miRNAs were spiked in total RNA of HeLa cell, and their Ct values were plotted as a function of their concentrations.

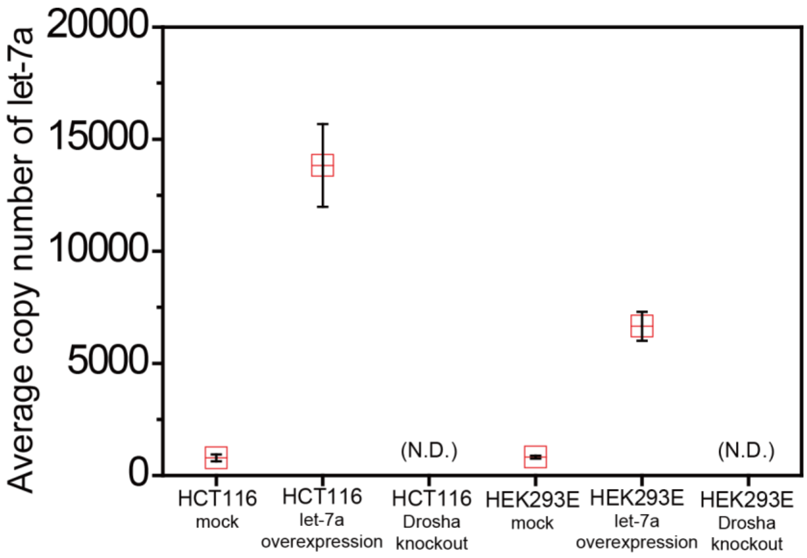

Supplementary Figure 7. Detection of dynamic changes of let-7a miRNA expression using Ago-FISH. Quantifications of let-7a miRNA of let-7a overexpression, and Drosha knockout cells were compared with those of mock HCT116 and HEK293E cells. To obtain each data point, three independent experiments were conducted. Data are mean ± SD.

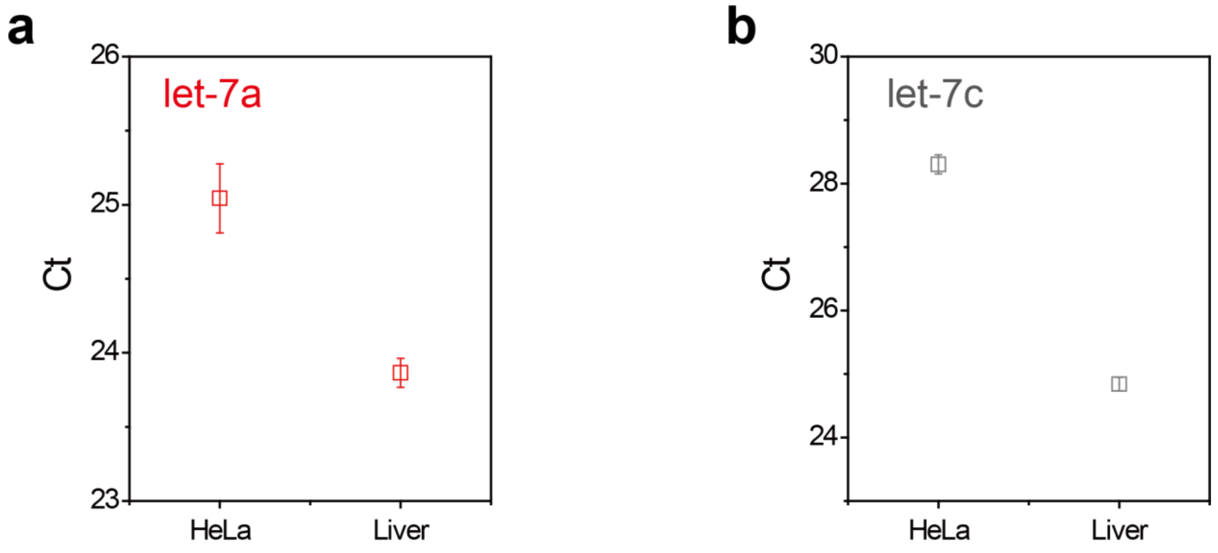

Supplementary Figure 8. miRNA quantification using qRT-PCR. Ct values for let-7a (a) and let-7c (b) in total RNA from HeLa cells and human liver tissue. Ct values are not normalized.

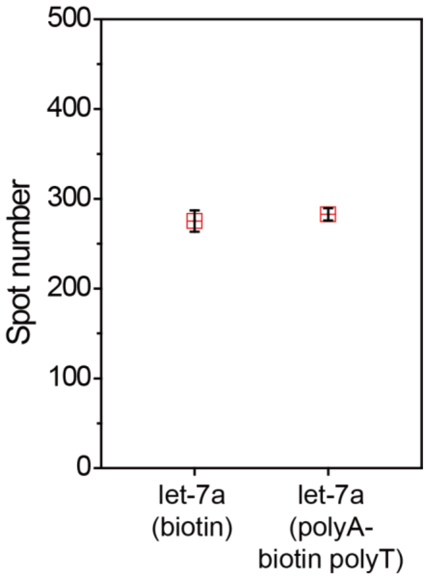

Supplementary Figure 9. Efficiency of poly(A) tailing followed by poly(T) hybridization. To obtain each data point, three independent experiments were conducted. Data are mean ± SD.

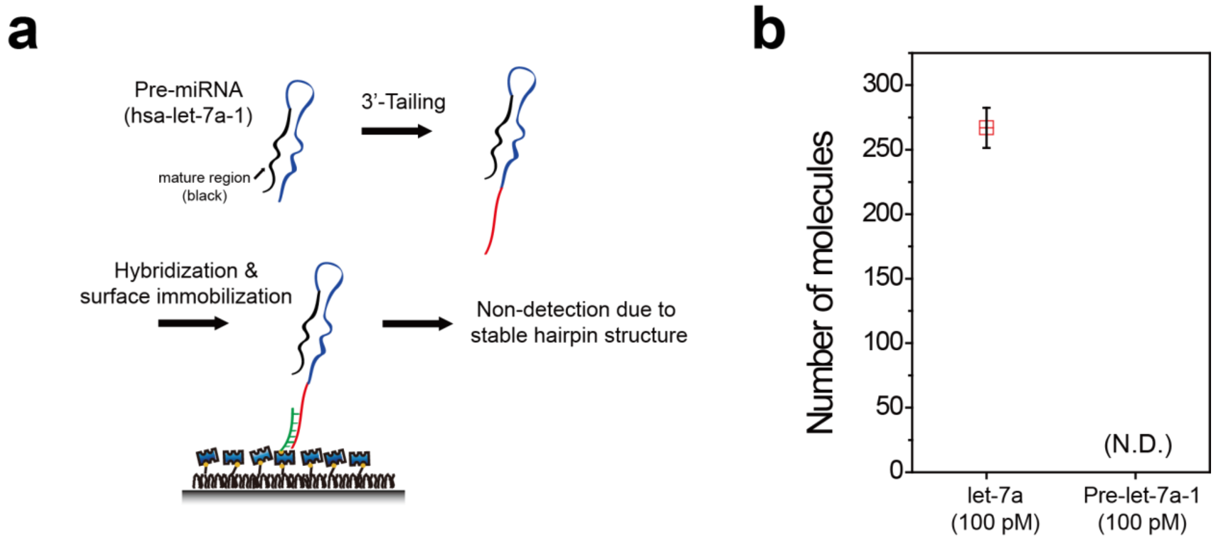

Supplementary Figure 10. No contamination of miRNA quantification by pre-miRNAs. (a) The experimental flowchart for the detection of pre-let-7a (Sequence information is available in Supplementary Fig. 1). (b) Comparison of let-7a and pre-let-7a quantification when the same concentration (100 pM) of target RNAs were immobilized. miRNA detection experiment was conducted at 30 ℃ where secondary structure of pre-miRNA is stable. Therefore, DNA probes cannot access the target region of pre-miRNA, and none of the pre-let-7a was detected. To obtain each data point, three independent experiments were conducted. Data are mean ± SD.
