# Supplementary material for: Quantification of purified endogenous miRNAs with high sensitivity and specificity
Source: Nat Commun. 2020 Nov 27;11:6033. doi: 10.1038/s41467-020-19865-9 (PMC7699633; doi:10.1038/s41467-020-19865-9)
Supplement: Supplementary file 2 — Reporting Summary [file 41467_2020_19865_MOESM2_ESM.pdf]

nature research

Corresponding author(s): Sungchul Hohng

Last updated by author(s): Nov 3, 2020

Reporting Summary

Nature Research wishes to improve the reproducibility of the work that we publish. This form provides structure for consistency and transparency in reporting. For further information on Nature Research policies, see our Editorial Policies and the Editorial Policy Checklist.

Statistics

For all statistical analyses, confirm that the following items are present in the figure legend, table legend, main text, or Methods section.

n/a	Confirmed	
☐	☒	The exact sample size (n) for each experimental group/condition, given as a discrete number and unit of measurement
☐	☒	A statement on whether measurements were taken from distinct samples or whether the same sample was measured repeatedly
☐	☒	The statistical test(s) used AND whether they are one- or two-sided<br>*Only common tests should be described solely by name; describe more complex techniques in the Methods section.*
☒	☐	A description of all covariates tested
☒	☐	A description of any assumptions or corrections, such as tests of normality and adjustment for multiple comparisons
☐	☒	A full description of the statistical parameters including central tendency (e.g. means) or other basic estimates (e.g. regression coefficient) AND variation (e.g. standard deviation) or associated estimates of uncertainty (e.g. confidence intervals)
☐	☒	For null hypothesis testing, the test statistic (e.g. F, t, r) with confidence intervals, effect sizes, degrees of freedom and P value noted<br>*Give P values as exact values whenever suitable.*
☒	☐	For Bayesian analysis, information on the choice of priors and Markov chain Monte Carlo settings
☒	☐	For hierarchical and complex designs, identification of the appropriate level for tests and full reporting of outcomes
☒	☐	Estimates of effect sizes (e.g. Cohen's d, Pearson's r), indicating how they were calculated

Our web collection on statistics for biologists contains articles on many of the points above.

Software and code

Policy information about availability of computer code

Data collection	All data were acquired by using custom software (Visual C++ 2010, Microsoft) which is a modified version of "SINGLE" software that was developed by the lab of Prof. Dr. Ha. The original version is available at: http://ha.med.jhmi.edu/resources/
Data analysis	Data were analyzed by custom codes(MATLAB(R2010a, The MatWorks), IDL(7.0, ITT), and Origin(8.0, OriginLab)). The scripts are available upon request.

For manuscripts utilizing custom algorithms or software that are central to the research but not yet described in published literature, software must be made available to editors and reviewers. We strongly encourage code deposition in a community repository (e.g. GitHub). See the Nature Research guidelines for submitting code & software for further information.

Data

Policy information about availability of data

All manuscripts must include a data availability statement. This statement should provide the following information, where applicable:

- Accession codes, unique identifiers, or web links for publicly available datasets
- A list of figures that have associated raw data
- A description of any restrictions on data availability

We have provided a full data availability statement in the manuscript.

Field-specific reporting

Please select the one below that is the best fit for your research. If you are not sure, read the appropriate sections before making your selection.

[x] Life sciences [ ] Behavioural & social sciences [ ] Ecological, evolutionary & environmental sciences

For a reference copy of the document with all sections, see nature.com/documents/nr-reporting-summary-flat.pdf

Life sciences study design

All studies must disclose on these points even when the disclosure is negative.

Sample size	Three or more independent experiments were conducted. The minimal number of data for bootstrap analysis is ~50. We analyzed more than 300 molecules for each data point to ensure the accuracy.
Data exclusions	In some cases, attempts to reproduce data failed because of the poor surface-passivation of microscope slides. These data were excluded and repeated on high-quality microscope slides.
Replication	To verify the reproducibility, three or more independent experiments were performed and analyzed. We confirm we can reproduce the data presented in this manuscript.
Randomization	In our study, sample allocation into the experimental group was unnecessary, therefore,randomization was not required in our study.
Blinding	Blinding was not relevant to our study, since grouping was not performed in our study.

Reporting for specific materials, systems and methods

We require information from authors about some types of materials, experimental systems and methods used in many studies. Here, indicate whether each material, system or method listed is relevant to your study. If you are not sure if a list item applies to your research, read the appropriate section before selecting a response.

Materials & experimental systems

n/a	Involved in the study	
[x]	[ ]	Antibodies
[ ]	[x]	Eukaryotic cell lines
[x]	[ ]	Palaeontology and archaeology
[x]	[ ]	Animals and other organisms
[x]	[ ]	Human research participants
[x]	[ ]	Clinical data
[x]	[ ]	Dual use research of concern

Methods

n/a	Involved in the study	
[x]	[ ]	ChIP-seq
[x]	[ ]	Flow cytometry
[x]	[ ]	MRI-based neuroimaging

Eukaryotic cell lines

Policy information about cell lines

Cell line source(s)	HeLa(human, ATCC)<br>MCF-7(human, ATCC)<br>HCT116(human, ATCC, genetically modified cells from V. N. Kim lab, see ref. 30)<br>HEK293E(human, ATCC, genetically modified cells from V. N. Kim lab)
Authentication	None of the cell lines have been authenticated.
Mycoplasma contamination	Cell lines were not tested for mycoplasma contamination but no indication of contamination was observed.
Commonly misidentified lines (See ICLAC register)	No commonly misidentified cell lines were used.
